# Ovarian neoplasia in adolescence: a retrospective chart review of girls with neoplastic ovarian tumors in Saudi Arabia

**DOI:** 10.1186/s13048-022-01033-w

**Published:** 2022-09-16

**Authors:** Lateefa AlDakhil, Asma Aljuhaimi, Mashael AlKhattabi, Saleh Alobaid, Rafif E. Mattar, Abdulaziz Alobaid

**Affiliations:** 1 Department of Obstetrics and Gynecology, King Saud University Medical City, King Saud University, PO box 245-634, Riyadh, 11312 Saudi Arabia; 2 King Saud Medical City, Riyadh, Saudi Arabia; 3 Department of Gynecology, Women’s Specialized Hospital, King Fahad Medical City, Riyadh, Saudi Arabia; 4 Alfaisal University, Riyadh, Saudi Arabia; 5 Department of Surgery, King Saud University Medical City, King Saud University, Riyadh, Saudi Arabia

**Keywords:** Gynecology, Adolescent, Fertility preservation, Ovarian tumor, Malignancy

## Abstract

**Background:**

Ovarian neoplasia in children and adolescents is a rare tumor. The diagnosis and management of such tumors is often difficult and delayed due to non-specific symptoms and low suspicion. Surgical management that preserves fertility and ovarian function should be the goal.

**Objective:**

This study aimed to review the clinical presentation, tumor characteristics, and management of Saudi Arabian adolescents.

**Methods:**

A retrospective chart review was conducted on adolescent girls aged 19 or less admitted to tow referral hospital in Riyadh, Saudi Arabia, diagnosed with adnexal mass over an 8 years’ period; patients who were older than 19 were excluded.

The data collected from patients’ charts included age, presenting symptoms, radiologic findings, type of surgery, specialist who performed the surgery, and histopathology of the tumors.

We classified patients according to age using the three WHO developmental stages: early adolescence (10–13 years old), middle adolescence (14–16 years old), and late adolescence (16–17 years old).

The statistical study used SPSS version 18.0 to determine the data’s frequency, distributions, and means (SPSS Inc., Chicago, IL).

**Results:**

We analyzed 164 patients, between 10 and 19 years old, admitted to two hospitals between 2009 and 2017. We found that 85% of these patients underwent surgery for adnexal mass removal, and 90.2% were symptomatic or emergency cases. The majority of our patients were post-menarche (96.95%), and were between the ages of 14 and 19. The most common surgical procedure for tumor removal was laparoscopic cystectomy (74.4%).

An adnexal mass with a solid component on ultrasound is the most commonly found indicator of malignancy. The majority of tumors were benign (32.3%). Germ cell tumors were the most common (68.7%) malignant tumor, and yolk sac tumors were the most common subgroup of germ cell tumors. When managed by a gynecologist, surgical intervention can be a successful method of preserving fertility.

**Conclusions:**

Our results confirm that the majority of neoplastic ovarian tumors in children and adolescents are benign, and surgical intervention can be used to maintain fertility, especially when managed by a gynecologist. This is one of the largest reported series and the first from our area.

## Background

Ovarian cancer is a fatal gynecologic malignancy, and due to the absence of early signs and screening regimens, it is discovered late in the illness’s clinical course. The global incidence varies. It has an age adjusted incidence of 6.3–12.1/100,000 women and is the seventh most common cancer diagnosis [1, 2]. It is reported as the fourth cause of death among women [3, 4]. There is an epidemiological variability of ovarian cancer among locations and ethnicity [5, 6]. Neoplastic ovarian tumors in children and adolescents are rare, and the reported incidence in this age group ranges from 0.9 to 2% of all tumor types [7]. Several studies have also reported an incidence of approximately 2.6 cases per 100,000 females per year in this age group [8, 9].

Although the majority of these ovarian tumors are benign, a small number (0.2%) are malignant. The malignant tumors mostly are treatable, and have high survival rates. However, these indolent malignant tumors tend to become aggressive when inadequately treated [8]. Thus, the main goal of management is to promote an excellent outcome while reducing morbidity and preserving fertility.

The data on ovarian tumor types in various parts of the world have reported that germ cell tumors predominate among children and that approximately 70% of such ovarian neoplasms occur in adolescents, with mature teratoma being the predominant type of benign tumor and dysgerminoma being the predominant type of malignant tumor [7].

A review of 2026 childhood ovarian tumors reported in studies from 1940 to 1993 found that 33% were malignant and 67% were benign. The most common childhood ovarian malignancy is dysgerminoma, which accounts for 9.5 to 11% of all childhood ovarian tumors and 24.5% of childhood ovarian malignancies [8]. It has been reported that the most common type of ovarian tumor reported has varied from place to place; however, data from Ghana showed that among a total of 67 cases, 44 (65.7%) are germ cell tumors. Burkitt lymphoma, not dysgerminoma, was the single most common malignant tumor of the ovary [10]. No study of histopathology results of ovarian neoplasia in adolescents reported in our area was found in the literature.

The treatment of ovarian neoplasia often involves monitoring the patient’s surgical success. The surgical treatment for ovarian neoplasia can be performed in two ways: either by removing the tumor alone (ovary-sparing surgery) or by removing the lesion and the entire ovary (oophorectomy). Although existing data supports ovary-sparing surgery for benign ovarian neoplasms, new research indicates that up to 50% of pediatric patients with benign tumors undergo an oophorectomy [11–13].

Due to the low number of cases reported in adolescents and children, both the diagnosis and management of ovarian tumors in this group pose many challenges. First, most of the reported data comes from tertiary centers, which are limited by their small sample sizes. Second, these data include a mixed population of children, adolescents, and young women, with the terms “children” and “adolescents” being used to describe a nonspecific variety of age groups ranging from birth to 21 years of age [10]. These classifications are inconsistent with the World Health Organization’s (WHO) definition of the term “adolescent;” namely, a person between 10 and 19 years old. Adolescence can be further subdivided into early adolescence (10- to 13-year age group), middle adolescence (14- to 16-year age group), and late adolescence (17- to 19-year age group) [14].

There have been no published studies looking into the findings in each group or whether they differ from early, middle, and late adolescence. There is no local data reported. Most reported data have small patient numbers (< 50). In this study, we examined cases of adolescents diagnosed with ovarian tumors who were managed and admitted in gynecology departments of two referral hospitals—King Fahad Medical City and King Khalid University Hospital—in Riyadh, Saudi Arabia.

## Results

### Patient characteristics

The charts of all patients were reviewed and ensured they met the criteria for data collection. The study included all girls aged 19 or younger and admitted for ovarian mass treatment; girls above this age were excluded. In total, we collected 164 patients aged between 10 and 19 years who underwent surgeries for adnexal mass removal. These patients were around 4.9% of all patients managed in both hospitals for an ovarian mass in the study periods (more than 85% of these patients were in the applicable age group). The median age of all patients was 16.83 years. A total of 159 (96.95%) patients were post-menarche, and five (3.04%) patients were pre-menarche (Table 1).

**Table 1:** Demographics characteristics of patients

Characteristics	Frequency (%)
Total patients (n)	164
Mean age (range), years old	16.83 (10–19)
Total pre-menarche girls (%), n	159 (96.95%)
Total post-menarche girls (%), n	5 (3.04%)
Mean body mass index (range), kg/m2	21.21
Age groups	3

Table 2 shows the distribution of tumors among the three-age group, the highest number of tumors was diagnosed in the late adolescent age group (17–21) with total of 100 (61%) cases, and the lowest is in the early adolescent (11–13) with only 12 cases. There was no difference among the three groups in regard of possibility of malignancy.

**Table 2:** Tumor distribution among adolescent age group

	Total patients	Benign N (%)	Malignant N (%)	Non-neoplastic	RR (95% CI)	***P***-value
**Early Age (11–13)**	12	4 (33.3)	1 (8.3)	7 {58.3%}	0.25 (0.03, 1.92)	0.1829
**Middle Age (14–16)**	59	15 (25.4)	4 (6.8)	40 {67.8%}	0.27 (0.09, 0.76)	0.0129
**Late Age (17–21)**	93	30 (32.3)	10 (10.7)	53 {57%}	0.33 (0.17, 0.64)	0.0010

The majority of patients were symptomatic (61%) at presentation, reporting mainly abdominal pain, and 32.3% were emergency cases presenting with torsion or rupture. The remainder of the patients were asymptomatic at presentation (Table 3), and none of the malignant tumors were an incidental finding.

**Table 3:** Clinical presentation and type of malignancy

	Total	Benign	Malignant	RR (95% CI)	***P***-value
**Symptomatic**	100	32 (32.0)	14 (14.0)	0.44 (0.25, 0.77)	0.0040
**Incidental/Asymptomatic**	11	6 (54.5)	0 (0)	0.08 (0.005, 1.22)	0.0689
**Emergency**	53	11 (20.8)	1 (1.9)	0.09 (0.01,0.68)	0.0195

Data is presented as a frequency (percentage) of the clinical presentation of all patients

*p <  0.01 therefore all values are significantly different

Pelvic ultrasound (US) was performed preoperatively as part of the initial work-up (Table 4). On the pelvic US, the mean tumor size was determined by measuring the longest diameter. For right-sided tumors, the mean diameter was 8.8 cm (0.3, 39.2) and for left-sided tumors, it was 10.4 cm. A significant association was not found between the size of the adnexa and malignancy histopathology. Some patients underwent further investigation by means of another imaging modality: 50 patients (30.4%) had abdominal CT scans, and 19 (11.5%) underwent magnetic resonance imaging.

**Table 4:** Ultrasound finding and type of tumor

**Tumor size**	**Total**	**Benign**	**Malignant**	**RR (95% CI)**	***P*-value**
**Greater 10** **(>** **10)**	46	13 (28.3)	2 (4.3)	0.15 (0.04, 0.64)	0.0104
**Equal or Less than 10 (< 10)**	85	15 (17.6)	5 (5.9)	0.33 (0.13,0.88)	0.0259
**Unknown**	33	N/A	N/A	N/A	N/A
**Tumor characteristic**	**Total**	**Benign**	**Malignant**	**RR (95% CI)**	***P*-value**
**Simple/ Null / N/A**	97	12 (12.4)	1 (1.03)	0.08 (0.01, 0.63)	0.0159
**Complex (solid component)**	13	3 (23.1)	10 (76.9)	3.33 (1.18, 9.39)	0.0228
**Solid**	3	0 (0)	1 (33.3)	3.01 (0.17, 53.7)	0.4554
**Multi-cyst/Multi-occulated**	25	9 (36.0)	2 (8.0)	0.22 (0.05, 0.93)	0.0390
**Dermoid**	25	25 (100)	0 (0)	N/A	N/A

*RR* relative risk, *95% CI* 95% Confidence interval

In terms of predicting the final histopathology results, complex and solid tumors have a higher relative risk for malignancy (Table 4). Among the surgical approaches utilized, conventional laparoscopy was used in 122 (74.4%) patients, single-port laparoscopy was used in 12 (7.3%) patients, and laparotomy was used in 30 (18.3%) patients. Almost all patients (163) underwent fertility-preserving surgery (> 98%). All surgical procedures were performed by gynecologic oncology or a general gynecology specialist, and the most common procedure was a unilateral cystectomy.

Of all ovarian neoplasms examined herein, 15 (9%) were malignant tumors, 49 (30%) were benign neoplasia, and 100 (61%) were functional (physiological) cysts (Table 5). The majority of cases (148 cases, 90%) were unilateral. Among the patients with unilateral tumors, there was no difference in the tumor side: 75 (50.7%) had right unilateral disease and 73 (49.3%) had left unilateral disease.

**Table 5:** Distribution of tumor types based on unilateral and bilateral histopathology

Histopathology (Type)	Unilateral	Bilateral	total	*P* value^*^
Non-neoplastic/ Physiological	91	9	100	< 0.01
Benign	43	6	49	< 0.01
Malignant	14	1	15	< 0.01

Data is presented as frequency (percentage) of cyst types based on the unilateral and bilateral histopathology of all patients

*p < 0.01 Therefore, all of the values are significantly different

Analysis of benign tumors showed that mature cystic teratoma was the most common form of benign histopathology (52.8%, *n* = 28), followed by serous cystadenoma (15%, n = 8), serous cystadenofibroma (15%, *n* = 8), mucinous cystadenoma (11.3%, *n* = 6), and endometrioma (5.7%, *n* = 3). Among malignancies, 65% of the patients with malignant tumors had stage 1 disease at diagnosis, and 85.7% of these patients received adjuvant chemotherapy.

In this cohort, germ cell tumors were the most common type of malignant tumor (56.24%, *n* = 9), and yolk sac tumors were the most common subgroup of germ cell tumors (37.5%, *n* = 6). Additionally, of all malignancy’s dysgerminoma were (12.5%, *n* = 2), mixed germ cell tumors (6.25%, *n* = 1) and other malignancies included Sertoli cell tumors (6.25%, *n* = 1), juvenile granulosa cell tumors (6.25%, *n* = 1), mucinous cystadenocarcinoma (25%, *n* = 4) and serous cystadenocarcinoma (6.25% *n* = 1).

## Discussion

Ovarian tumors in adolescents’ girls represent a rare tumor with heterogeneous histopathologic entities and there are less than 50 reported cases [15]. The literature for the management and outcome of this group of patients is limited. This series represents a relatively large group of patients.

In our review, the most common presenting symptom was abdominal pain. Ninety percent of the patients were symptomatic, reporting acute or chronic abdominal pain as the most common symptom, and 32.9% of these patients were emergency cases, presenting with rupture or torsion.

Other data reported dysmenorrhea, and palpable abdominal mass as their most common symptoms [3, 10].

Clinical presentation had no significant impact on our histopathological results that are consistent with other published data [16–18]. The majority of our patients had unilateral disease (90%), which aligned with previous reported data [6, 9, 19]. The management of these cases’ ultrasound examinations is the initial step that showed the only significant factor correlating with malignancy risk is the presence of a solid tumor or complex component; there was no significant correlation between size and risk of malignancy that was consistent with other reports [20]. There is reported data that found a significant correlation between malignancy and tumor size where some data used 10 cm and others used a 12 cm cutoff; however, these data focused on ovarian masses in children and younger adolescent [21–23]. The ovarian volume or mass could be a better predictor of risk of malignancy, as previously reported [22, 24]. This could be due to problems of accuracy with tumor diameter measurements on US. The finding of a solid component on US was the most important indicator of malignancy, which was consistent with our finding [25, 26]. The need for extra imaging was limited to a small number of patients, and the US report usually recommended extra studying or there is suspicion of metastatic disease. as US imaging has high sensitivity (76%) and specificity (100%) [21].

In our series, ovarian tumors in adolescents accounted for less than 5% of all ovarian tumors, and the majority of these cases were functional ovarian cysts. Reports from China found that 5% of all ovarian tumors in china occurred in children and adolescents [27, 28]. Conversely, reports from Ghana and Nigeria showed a higher rate of ovarian tumors (8–9%) in children and adolescents [10, 29].

In looking into the distribution of the prevalence of ovarian tumors and the risk of malignancy among the three different stages of adolescence, we found that the majority of tumors occur in the late adolescent group with no difference in risk of malignancy among the three age groups.

Germ cell tumors were the most common group of neoplastic tumors in both malignant and benign tumors. Mature teratomas (52.8%) in benign tumors and, yolk sac tumors (37.5%), and dysgerminomas (12.5%) of malignant tumors. This finding is in line with the study results that Xac and Jetelina [30] proposed. Our study showed that the distribution of germ cell tumors showed that yolk sac tumors rather than dysgerminoma were the most common type of malignancy, as reported in the other studies (up to 70%) from USA and Europe [8, 9]. Other types of histopathology (Burkitt’s lymphoma) were reported in Nigeria and Ghana [10, 29].

The overall goals of management in these cases is alleviation of symptoms and/or treating the malignancy while preserving ovarian function as much as possible. These cases are usually managed by gynecologist either (general or oncology) or by surgeon (pediatric or general). One clinical study showed that the rate of ovarian preservation is significantly higher if a gynecologist performed the tumor removal surgery. Bristow [31] reported that the rate of ovarian preservation was 62.3% for patients managed by a gynecologist in contrast to 20.7% for patients managed by a pediatric surgeon. Consistent with this finding, the analysis of our series revealed that the ovarian preservation rate was above 90% for patients managed by a gynecologist only, while a similar study reported an ovarian preservation rate of only 61% when managed by gynecologist and surgeons [32]. Other studies indicated the positive correlation between preservation of the ovary when a gynecologist managed this surgery compared to a pediatric surgeon [26]. Our data investigated a relatively large group of patients (164), which is one of the largest reported series in this age group and the only data from our area.

## Limitations

Ovarian tumors in children and adolescents represent a heterogeneous group of histopathological entities. This study has several limitations, the most notable of which is its retrospective nature. Specifically, there are unique challenges to data collection and research in retrospective studies. In this analysis, our results were subject to issues involving recall bias, missing data, and a lack of patient follow-up. Retrospective cohort studies require larger population sizes because some outcomes are rare and prone to misclassification bias [33].

## Conclusions

Ovarian tumors in young girls and adolescents represent a heterogeneous group of histopathological entities. Although these tumors are uncommon, there is a 10% risk one could be malignant. In this study, a large sample of children and adolescents diagnosed with ovarian tumors and treated in Saudi Arabia was analyzed. Our results show that the most significant indicator of malignancy is an adnexal mass with a solid component on ultrasound rather than the tumor’s diameter alone. The highest number of tumor were in the late stage [17–21] and the distribution of tumor types among the three stages of adolescence is similar. The most common presenting symptom of adnexal masses was abdominal pain. Ninety percent of the patients had unilateral disease, and in this group, there was no significant difference in the incidence of tumors on the left or right side. Our data revealed a high success rate with ovarian preservation when a gynecologist managed the surgery. The distribution of germ cell tumors was different from that in cohort studies, in which yolk sac tumors were the most frequent type of malignancy. Among them, malignant ovarian masses are rare but treatable tumors, and fertility preservation efforts can be successful.

## Materials and methods

### Study design and data collection

We conducted a retrospective chart review of 164 adolescent females aged 10 to 19 years with adnexal masses who were managed and admitted to two different referral hospitals in Riyadh, Saudi Arabia (King Fahad Medical City and King Khalid University Hospital) in the 8 years from January 1, 2009 to December 31, 2017. Interviews were conducted to acquire demographic information and informed permission from all pediatric patients and their parents. The following data were collected: the age of the adolescents at the time of presentation, the patient’s clinical presentation, tumor location, ultrasonography findings, and histology. We gathered imaging reports using transabdominal ultrasonography (US). We classified patients according to their ages using the three WHO developmental stages: early adolescence (10–13-years-old), middle adolescence (14–16-years-old), and late adolescence (16–17-years-old). Each ovarian ultrasound finding was classified as simple, complicated, or solid [15]. The surgical procedure was conducted by the general gynecology or gynecology oncology team, and was done as an emergency or on an elective basis. In the instance of ovarian torsion, the initial step is to untwist the ovary; the surgeon will then determine whether to remove or preserve the ovary based on its macroscopic appearance after detorsion. Treatment methods for cystic ovarian lesions include ovarian sparing surgery (cystectomy) or laparoscopic oophorectomy [16].

### Staining methods

Pathologists specializing in gynecologic pathology at both hospitals in Riyadh, Saudi Arabia, analyzed tissue specimens following the WHO’s International Classification of Ovarian Tumors and the International Federation of Gynecology and Obstetrics (FIGO) staging criteria [17].

### Statistical analysis

The statistical study used SPSS version 18.0 to determine the data’s frequency, distributions, and means (SPSS Inc., Chicago, IL).

## Data Availability

Data are available from Lateefa Othman Aldakhil upon request and with permission of King Fahad Medical City and King Khalid University Hospital.

## Abbreviations

WHO: World Health Organization
FIGO: International Federation of Gynecology and Obstetrics
US: Pelvic Ultrasound
